# Wanted, a Dog Hospital

**Published:** 1884-04

**Authors:** 


Wanted, a Dog Hospital.—This is a hospital era. We have hospitals for every class of human disease; hospitals for curables and incurables, hospitals for emergencies, and hospitals for convalescents.

This present exacerbation of hospital organization, however, limits itself to man, who is, of all creation, alone vile. We would make a plea for some of the lower animals. We need a hospital for dogs, cats, and the small domestic animals of all kinds. The amount of money in pet dogs, watch-dogs, and house-dogs in this city and suburbs is very great. Yet there is no good place where sick dogs can be well taken care of. To be sure there are the veterinary hospitals, but they are no better than stables, and are not suitable for small and delicate dogs.

---

A PLATE, which, owing to an accident, was not ready for insertion in the preceding number of the JOURNAL, will be found accompanying the present one. It contains illustrations referred to in the article on the "Fundus of the Eye of the Horse," by Dr. Berlin.

